# Working for Food Shifts Nocturnal Mouse Activity into the Day

**DOI:** 10.1371/journal.pone.0017527

**Published:** 2011-03-30

**Authors:** Roelof A. Hut, Violetta Pilorz, Ate S. Boerema, Arjen M. Strijkstra, Serge Daan

**Affiliations:** Chronobiology Unit, Center for Behavior and Neurosciences, University of Groningen, Groningen, The Netherlands; Vanderbilt University, United States of America

## Abstract

Nocturnal rodents show diurnal food anticipatory activity when food access is restricted to a few hours in daytime. Timed food access also results in reduced food intake, but the role of food intake in circadian organization *per se* has not been described. By simulating natural food shortage in mice that work for food we show that reduced food intake alone shifts the activity phase from the night into the day and eventually causes nocturnal torpor (natural hypothermia). Release into continuous darkness with *ad libitum* food, elicits immediate reversal of activity to the previous nocturnal phase, indicating that the classical circadian pacemaker maintained its phase to the light-dark cycle. This flexibility in behavioral timing would allow mice to exploit the diurnal temporal niche while minimizing energy expenditure under poor feeding conditions in nature. This study reveals an intimate link between metabolism and mammalian circadian organization.

## Introduction

Mice and rats are nocturnal, but their activity shifts to the day when food access is restricted to a few hours in daytime. In such restricted schedules the food received is limited both in time and in quantity (typically 60–70% of normal daily food intake). This procedure induces food anticipatory activity (FAA) a few hours before the food is delivered [1]. FAA is thought to be the behavioral output of a separate circadian oscillator called the food entrainable oscillator (FEO) which is entrained by periodic food availability and capable of driving peripheral [2], [3] and behavioral rhythms [4]. Under these conditions the FEO drives the activity rhythm independently of the suprachiasmatic nucleus (SCN), which controls nocturnal activity under standard *ad libitum* conditions [5], [6]. The anatomical substrate of the FEO remains elusive in spite of intense investigation and debate [4], [7]–[23].

In nature, food availability is hardly periodic to most rodents, and diurnal activity has often been observed in nocturnal animals [24]–[26]. A crucial difference between lab and field is that animals in the field have to work (*i.e.* spend time and energy) to obtain food. To study the effect of reduced food intake on circadian organization, we simulated natural food scarcity by having mice work for their food at increasing levels of workload under a 12 h light - 12 h dark (LD) cycle, without restricting food access to a specific time of day.

## Results

**Figure 1:**
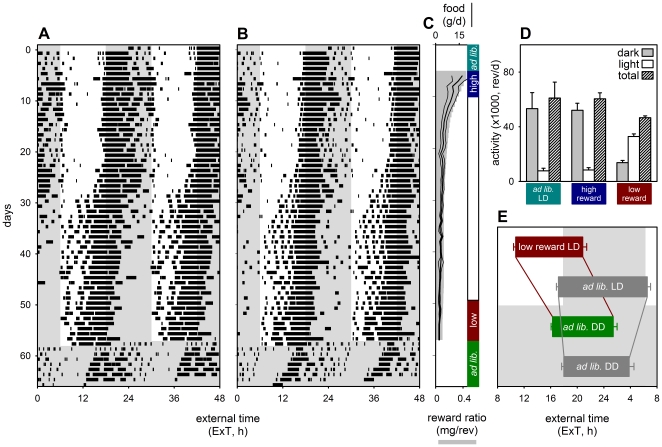
Low food reward ratio induces diurnal activity, with minor effects on daily activity levels and circadian pacemaker phase. (**A, B**) Two example actograms show the gradual shift from nocturnal to diurnal wheel running. (**C**) Gradual reward ratio reduction (grey bars) reduces total food obtained (line, in g/d; thin lines indicate s.e.m.). (**D**) Total activity per 24 h was slightly reduced but shifted largely to the day when the reward ratio was reduced (red compared to blue and cyan). The immediate shift to the previous dark phase after release in DD with *ad libitum* food indicates normal entrainment of the circadian pacemaker to the previous LD cycle (**A, B**; days 59–66). (**E**) Average activity onsets and offsets for working mice during the last 8 days under LD (red bar) and first 8 days in DD (green bar), and for the *ad libitum* control mice (grey bars) during the same time intervals.

Reducing food reward per unit workload (i.e. wheel running in mammals or perch hopping in birds) reduced daily food intake (Fig. 1C) as described in other species [28]. As a result mice spontaneously shifted their activity to the light phase of the day (Fig. 1A–B). This procedure increased activity in daytime while total activity per 24 h remained stable or was slightly reduced (Fig. 1D). Detailed comparison of activity onsets and offsets during the last 8 days in LD and the first 8 days in DD shows that low reward ratios advance activity onsets by 5.5 h (paired T-test, p<0.0001) and offsets by 4.6 h (paired T-test, p = 0.0003) relative to the estimated phase of the main circadian pacemaker during entrainment (as extrapolation from activity patterns after release in DD with *ad libitum* food; Fig.1E red vs. green bars). Control mice on *ad libitum* food access throughout the experiment did not show advanced activity onsets in LD but rather delayed offsets (paired T-test: onsets, 0.8 h advanced, p = 0.08; offsets, 2.7 h delayed p = 0.01; Fig.1E grey bars). Low reward ratio advanced activity onsets in LD by 6.4 h (p<0.0001) and offsets by 9.7 h (p<0.0001) when compared to control mice (Fig.1E, red vs. top grey bar). After release in DD and *ad libitum* food conditions, the estimated phase of the circadian pacemaker during entrainment was 1.7 h (p = 0.0004) advanced for the onset of activity and 2.4 h advanced for the offset of activity (green vs. grey bar) when compared to the *ad libitum* control mice. Taken together, these data indicate that reduced food intake shifts activity from the dark phase into the light phase. Upon release into DD with food available *ad libitum*, activity patterns instantaneously reverted to the previous dark phase (Fig. 1A, B). This may suggest that the circadian pacemaker had remained entrained to the LD cycle, albeit with a slightly advanced phase relative to that of mice kept on *ad libitum* food supply throughout the experiment (Fig. 1E).

**Figure 2:**
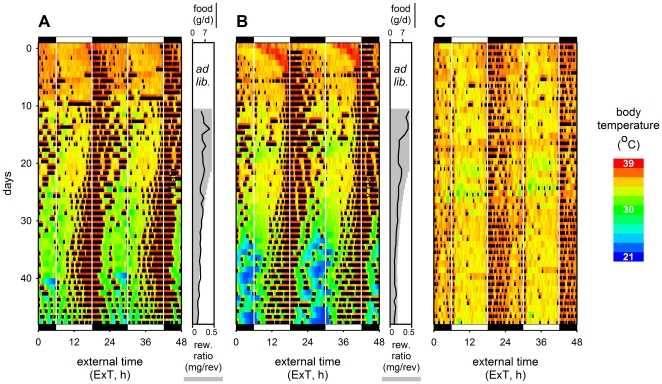
Reward reduction causes diurnal activity and eventually reduces body temperatures. (**A**, **B**) Two example actograms and body temperature patterns of mice under a reduced reward ratio protocol (grey bars) and (**C**) a control mouse kept under *ad libitum* conditions. Low daily food rewards (side panels, black lines) eventually elicited torpor response (blue colors) after activity patterns shifted to the light (see also Fig. S3). No evidence of torpor was found in any of the control mice. Black and white bars indicate the light-dark cycle to which the mice were exposed throughout the experiment.

Daily rhythms in core body temperature (T_b_) were associated with activity both in mice that work for their food and in *ad libitum* fed mice (Fig. 2). High T_b_ episodes gradually shifted to the light phase when reward ratio was reduced in mice working for food (Fig. 2A–B), but remained in the dark phase in control mice (Fig. 2C). While resting T_b_ rarely approached 30°C in control mice, mice under low reward ratio gradually reduced resting T_b_, eventually resulting in daily torpor well below 30°C (Fig. 2, Fig. S1) [29]. In some mice activity shifted to the light phase well before torpor occurred (Fig. S2), indicating that the behavioral shift is not caused by the occurrence of torpor (Fig. 2B).

Unlike control mice that show lowest T_b_ always during the light phase, lowest T_b_ in mice with a low reward ratio occurred in the last part of the night (Fig. 2A–B). The timing of torpor would thus coincide with the coldest phase of the daily cycle under natural conditions. This may have functional significance since at that time of day burrow retreat and torpor will have larger energetic benefits.

**Figure 3:**
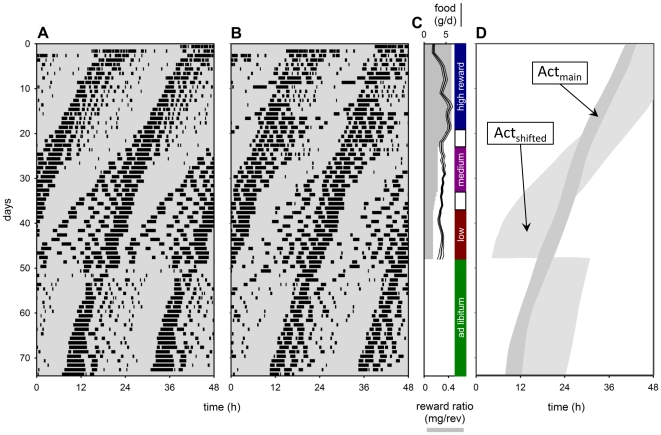
Reduced reward ratio modifies free-running circadian activity patterns in continuous dim light (DD). (**A**, **B**) Two examples of free-running activity rhythms in mice that work for food are shown in double plotted actogram format. (**C**) Gradual reward ratio reduction (grey bars) and food obtained (line, in g/d; thin lines indicate s.e.m.) shifted circadian activity patterns. (**D**) Graphical interpretation of observed activity patterns where a shifted activity component (Act_shifted_, light grey area) is discerned from a non-shifted main activity component (Act_main_, dark grey area). Four different work levels were applied: high reward ratio (C, blue bar), medium reward ratio (C, purple bar), low reward ratio (C, red bar), and *ad libitum* (C, green bar).

To test whether the shift in activity patterns from nocturnal to diurnal depends on the presence of a light dark cycle, we applied the work for food protocol in mice kept under continuous dim red light (DD, Fig. 3A–D). Here we identified a main activity component (Act_main_), which seems unaffected by the work for food protocol, and a less intense activity component (Act_shifted_) that followed the main activity component under high reward ratio and *ad libitum* conditions. Act_shifted_ component shifted and preceded the main activity component under medium and low reward ratio conditions (Fig. 3D). When mice under low reward ratio conditions were fed *ad libitum*, the preceding Act_shifted_ component immediately shifted back to its normal phase position and followed Act_main_. This observation is paralleled by the immediate return from diurnal to nocturnal activity patterns when working mice in an LD cycle were placed on *ad libitum* food access (Fig. 1. A–B).

## Discussion

Our data indicate that the output of the SCN driving activity cycles is modified downstream. Since the activity pattern gradually shifts towards diurnality when energy intake is reduced (Fig. 1A–B; 2A–B), it is plausible that this activity pattern is controlled by a slave oscillator that changes its phase position with respect to the SCN.

Activity-rest oscillators outside the SCN have been demonstrated both in temporally restricted fed animals (FEO [4]) and in rodents given access to low concentrations of methamphetamine in the drinking water (methamphetamine sensitive circadian oscillator, MASCO [30], [31]. The FEO, the MASCO, and the activity regulating oscillator presented here are all expressed under modified metabolic conditions. They may very well turn out to rely on the same physiological mechanism and anatomical structure.

**Figure 4:**
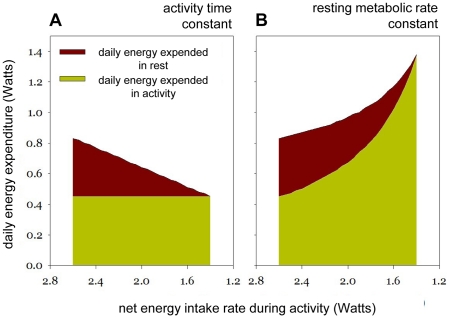
Energy budget calculation for two strategies to cope with reduced energy intake during foraging activity. Using known values for energy expenditure during rest and activity (see Text S1 - Model Calculations) we illustrate how mice may cope with reduced energy intake either by (**A**) reducing resting metabolic rate while duration of daily foraging activity remains constant (strategy A), or by (**B**) expanding daily foraging while metabolic rates during rest and activity remain constant (strategy B). With decreasing daily energy intake during activity, strategy A predicts a gradual reduction in body temperature during the rest phase, and reduced daily energy expenditure (DEE), while strategy B leads to a gradual increase in activity duration and DEE. Both strategies reach a limit when net energy intake during foraging drops below ∼1.4 Watts.

Reduced reward ratios in mice that run their wheel to obtain food resemble natural conditions, where travelling distance between food patches increases when food becomes scarce [29]. Our data may reveal the strategy that mice adopt to cope with reduced energy intake under natural conditions. Two alternative strategies to maintain energy balance under such conditions can be hypothesized (Fig. 4): A) foraging activity time per day remains constant and mice reduce energy expenditure during rest to match reduced energy intake; B) resting metabolic rate remains constant and mice increase foraging time per day to balance daily intake and expenditure. Both strategies were quantified and graphically represented (Fig. 4) using measures of energy expenditure from previous work (see Text S1 – Model Calculations). Although one might expect a combination of these strategies, the normal activity levels (Fig. 1) and the reduction in body temperature (Fig. 2) indicates that CBA/CaJ mice that work for food follow strategy A to maintain their energy balance when energy intake is reduced.

These results provide novel insight to the current debate on the FEO, in which mice show diurnal (food anticipatory) activity when feeding is restricted during a few hours in the light phase. This restricted diurnal meal timing is thought to uncouple activity rhythms from the circadian pacemaker (SCN) signal [4], [32]. The timed food restriction protocol used to study entrainment of the FEO carries the confounding risk that two variables are manipulated simultaneously: meal timing and reduced food intake (∼70–75% for 3 h food access in mice; [33]). Mendoza et al. [34] showed that diurnal activity occurs also when 6 short meals are presented at 4 h intervals, a procedure which still involves imposed meal timing. Mice in the present study chose themselves when to work for their food, indicating that reduced food intake alone, and not meal timing, was crucial to the occurrence of diurnal activity. Diurnal activity patterns in house mice were recently observed to persist for months under semi-natural conditions with competition for food access [35]. This diurnal behavior would allow mice to forage during the warmer part of the day and to time rest and hypothermia (torpor) at the end of the night. This temporal niche switching would minimize daily energy expenditure, possibly at the cost of increased predation risk during the day. The ‘work for food’ paradigm presented here reveals adaptive flexibility in circadian organization and a strong link between metabolism and the circadian system in an intact mammal. It avoids possible confounding effects of food timing and rationing, that are inherent to the classical food entrainment protocol. Spontaneous diurnal activity elicited in mice that work for food may open new approaches to describe an alternative circadian activity-rest oscillator, and will lead to a better understanding of temporal organization of behavior under natural conditions.

## Methods

All animal experiments were approved by the University of Groningen ethical committee (DEC 5011, 5114, 5454) and carried out following (inter-) national animal welfare regulations.

Adult male mice (CBA/CaJ; Jackson Laboratory, Bar Harbor, Maine, USA) were kept under standard laboratory conditions (see Text S1 – Detailed Methods). Sample sizes were as follows: Fig. 1&2: control n = 12, experimental n = 12; Fig. 3: n = 12. Running wheel activity was recorded continuously and stored in 2-min bins. Onsets and offsets of activity (Fig. 1E) were determined over 8 days just before the switch to DD or right after the switch to DD following the procedure previously described [27]. In the work for food protocol, a small 45 mg food pellet (F0165; Bio-Serv, Frenchtown NJ, USA) was delivered in the mouse cage after a set number of wheel revolutions using computer controlled pellet dispensers (Med Associates Inc., St.Albans VT, USA). During the ‘work for food’ protocol, mice started on a high reward schedule (100 revolutions/pellet). This generated essentially an *ad libitum* food condition, because excess pellets were observed in each individual kept under these conditions. During the reward reduction phases of the experiment, workload was increased daily by an extra 10–20 revolutions per pellet up to 200 revolutions per pellet (medium reward schedule) or 300–400 revolutions per pellet (low reward schedule). Throughout the experiment body mass was closely monitored at least every 3^rd^ day at different times of day and high workload levels were individually titrated to keep the mice above 18 grams and 75% of their initial body mass.

Body temperature was measured by customised temperature loggers (Thermochron iButton, DS1922L, Maxim Integrated Products Inc., Sunnyvale CA, USA) implanted in the abdominal cavity of the mice and set to a sampling rate of once every 20 minutes (see Text S1 – Detailed Methods).

## Supporting Information

Text S1 Supporting Information for ‘Working for food shifts nocturnal mouse activity into the day’.
(DOC)

Figure S1 **Reduced food reward affects body temperature patterns.** Body temperature patterns of two representative animals with gradually reduced food reward (**A**, **B**) and an *ad libitum* control animal (**C**). Body temperature occurring during the rest phase is reduced, eventually leading to torpor. Data are from the same animals as presented in Fig. 1 (panel identifiers correspond).
(TIF)

Figure S2 **Reduced food reward shifts activity rhythms before torpor occurs.** This example shows that some animals in the experiment presented in Fig. 2 and Fig. S1 clearly showed a shifted activity rhythm well before torpor occurs. The opposite (torpor occurs before the activity rhythm is shifted) was never observed. These data indicate that torpor is not necessary to shift the activity rhythms into the day. It is rather the shifted activity rhythms that allow torpor to occur in the dark phase.
(TIF)
