# Supplementary material for: Working for Food Shifts Nocturnal Mouse Activity into the Day
Source: PLoS One. 2011 Mar 30;6(3):e17527. doi: 10.1371/journal.pone.0017527 (PMC3068156; doi:10.1371/journal.pone.0017527)
Supplement: Text S1 — Supporting Information for ‘Working for food shifts nocturnal mouse activity into the day’. (DOC) [file pone.0017527.s001.doc]

**SI Text**

**Supporting Information for**

**Working for food shifts nocturnal mouse activity into the day**

Roelof A. Hut, Violetta Pilorz, Ate S. Boerema, Arjen M. Strijkstra, Serge Daan

**Detailed Methods**

Adult male mice (CBA/CaJ; Jackson Laboratory, Bar Harbor, Maine, USA) were kept in translucent cages (12.5 x 12.5 x 20 cm). They were provided with running wheels (14 cm diameter) and water *ad libitum*. Cages were placed in a climate controlled room (21°C, Rel.Hum. 60%) under either continuous incandescent dim red light (< 1 lux) or a 12-h:12-h light-dark cycle using white fluorescent tube lights (Philips TLD 58W/840) providing 76 lux (SD=20 lux) at the level of the cage.

Activity onsets were defined as those time points where activity in a 1 h running mean became higher than the average activity level in a running mean with the time base of the circadian cycle as determined by periodogram analysis over the same data. Offsets were determined when 1 h running mean activity levels became lower than the activity level in a running mean with the time base of the circadian cycle as determined by periodogram analysis over the same data [1].

Implanted temperature loggers (Thermochron iButton, DS1922L, Maxim Integrated Products Inc., Sunnyvale CA, USA) were customized by taking the loggers from their metal jacket, sealing them in plastic foil, and coating them with Elvax (Minimitter, Bend OR, USA) followed by 2 h 2% glutar-aldehyde sterilisation, three rinses with sterile saline, overnight incubation in 1 million IU/30ml Na-penicillin, followed by 2 rinses with sterile saline. Weight of the encapsulated temperature loggers was ~2 g. Before abdominal logger implantation, animals were operated under isoflurane (Abbott Laboratories Ltd., Kent, UK) inhalation anesthesia (2%) followed by 1mg/kg finidine analgetics post-operation treatment. After operation animals were closely observed during a week recovery period.

**Model calculations of two alternative energetic strategies in mice to cope with reduced net energy intake (see Fig.4)**

Model solutions are examples using parameter values from other mouse strains, not necessarily matching the body mass and activity patterns of the strain used in our experiments. They aim to demonstrate the basic qualitative principle, not to yield specific quantitative predictions for the present experimental data.

For simplicity we assume that a mouse is either foraging or resting, such that the fraction of time spent resting (R) equals 1-A, if A is the time spent actively foraging. In order to stay energetically in balance, the daily metabolizable (net) energy intake M_d_ (Watt) has to match the daily energy expenditure (DEE), here indicated as E_d_ (Watt).

M_d_ equals A times the net intake rate during foraging m_a_ (Watt). E_d_ equals the sum of the rates of energy expenditure during foraging (e_a_) and during rest (e_r_), each weighted by the fraction of time spent in these two states. Thus maintaining energy balance simply means:

A*m_a_ = A*e_a_ + (1-A)*e_r_  (equation 1)

Food abundance to a mouse is reflected in its net rate of metabolizable energy intake (m_a_) during activity (foraging). When m_a_ decreases due to poorer conditions, energy balance can be maintained by changing A or e_r_. We assume that e_a_ cannot be decreased without simultaneously jeopardizing m_a_ further, and we assume e_a_ is a constant.

In our example simulations of the two strategies, we use the following realistic values. Under *ad libitum* feeding conditions, at 20°C, a mouse spends 72.3 kJ/day (DEE), or 0.837 Watt [2]. Its RMR is 0.571 Watt [2]. The time active is 7.7 h/day or A= 0.321 [3]. The contribution of rest to DEE is then 0.571*(1-0.321)*24*60*60= 33.5kJ/day, or 0.388 Watt. Hence, the contribution of activity to DEE is 0.837-0.388= 0.449 Watt. This yields a figure for the metabolic rate during activity of 0.449/0.321 = 1.399 Watt. The metabolizable energy intake during activity is thus estimated at 0.837/0.321 = 2.607 Watt.

In summary, the parameter values for the model calculations were as follows:

Active fraction of the day in ad lib: A = 0.321

Energy expenditure during activity: e_a_ = 1.399 Watt

Contribution of activity to DEE: E_a_ = A*e_a_ = 0.449 Watt

Rest fraction of the day: R = 1-A = 0.679

Energy expenditure during rest: e_r_ = 0.571 Watt

Contribution of rest to DEE: E_r_ = (1-A)*e_r_ =0.388 Watt

Total daily energy expenditure DEE: E_d_ = E_a_ + E_r_ = 0.837 Watt

Metabolizable energy intake during activity: m_a_ = 2.607 Watt

While the metabolizable energy intake rate m_a_ is deduced from its *ad lib* value we can now solve equation 1 for two different strategies:

*Strategy A: Activity time A is kept constant at 0.321:*

The unknown variable e_r_ can be solved from the equation 2:

e_r_ = A * (m_a_ - e_a_) / (1-A) (equation 2)

Thus, e_r_ drops linearly with decreasing m_a_ (Fig.4A). There are only positive solutions for m_a_>e_a_ = 1.399 Watt.

*Strategy B: Resting metabolic rate e_r_ is kept constant at 0.571*

The unknown variable A can now be solved from the equation 3:

A = e_r_/(m_a_ – e_a_ + e_r_) (equation 3)

Thus, A increases hyperbolically with decreasing m_a_ (Fig.4B). The limit to A obviously is 1.0. At this limit m_a_ = e_a_. This is the same as for strategy A.

**References**

1. Spoelstra K, Albrecht U, van der Horst GT, Brauer V, Daan S (2004) Phase responses to light pulses in mice lacking functional per or cry genes**.** J Biol Rhythms 19**:** 518-529.

2. Vaanholt LM, De JB, Garland T, Jr., Daan S, Visser GH (2007) Behavioural and physiological responses to increased foraging effort in male mice**.** J Exp Biol 210**:** 2013-2024.

3. Vaanholt LM, Garland T, Jr., Daan S, Visser GH (2007) Wheel-running activity and energy metabolism in relation to ambient temperature in mice selected for high wheel-running activity**.** J Comp Physiol B 177**:** 109-118.
