# Supplementary figures and images for: Working for Food Shifts Nocturnal Mouse Activity into the Day
Source: PLoS One. 2011 Mar 30;6(3):e17527. doi: 10.1371/journal.pone.0017527 (PMC3068156; doi:10.1371/journal.pone.0017527)

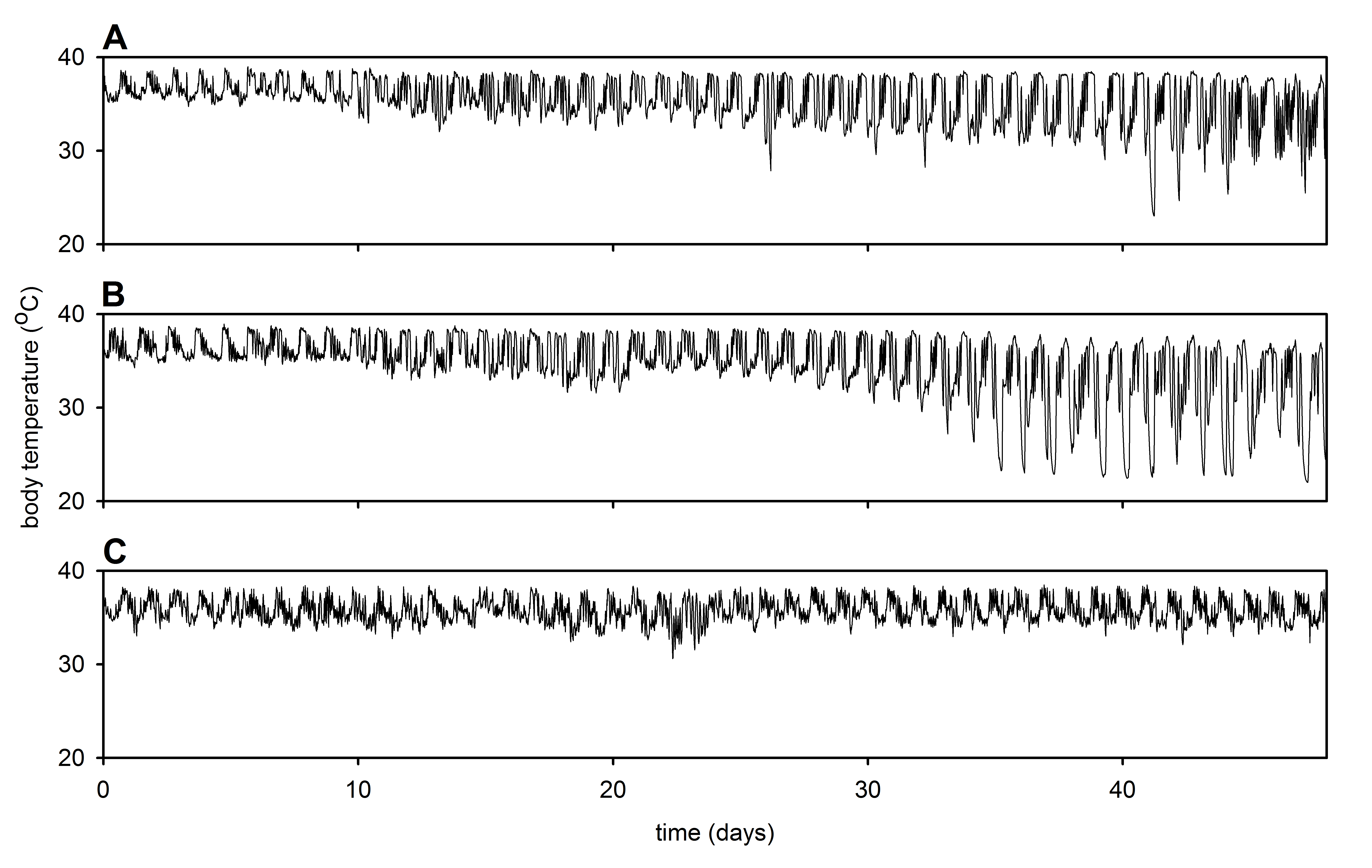

Supplement: Figure S1 — Reduced food reward affects body temperature patterns. Body temperature patterns of two representative animals with gradually reduced food reward (A, B) and an ad libitum control animal (C). Body temperature occurring during the rest phase is reduced, eventually leading to torpor. Data are from the same animals as presented in Fig. 1 (panel identifiers correspond). (TIF) [file pone.0017527.s002.tif]

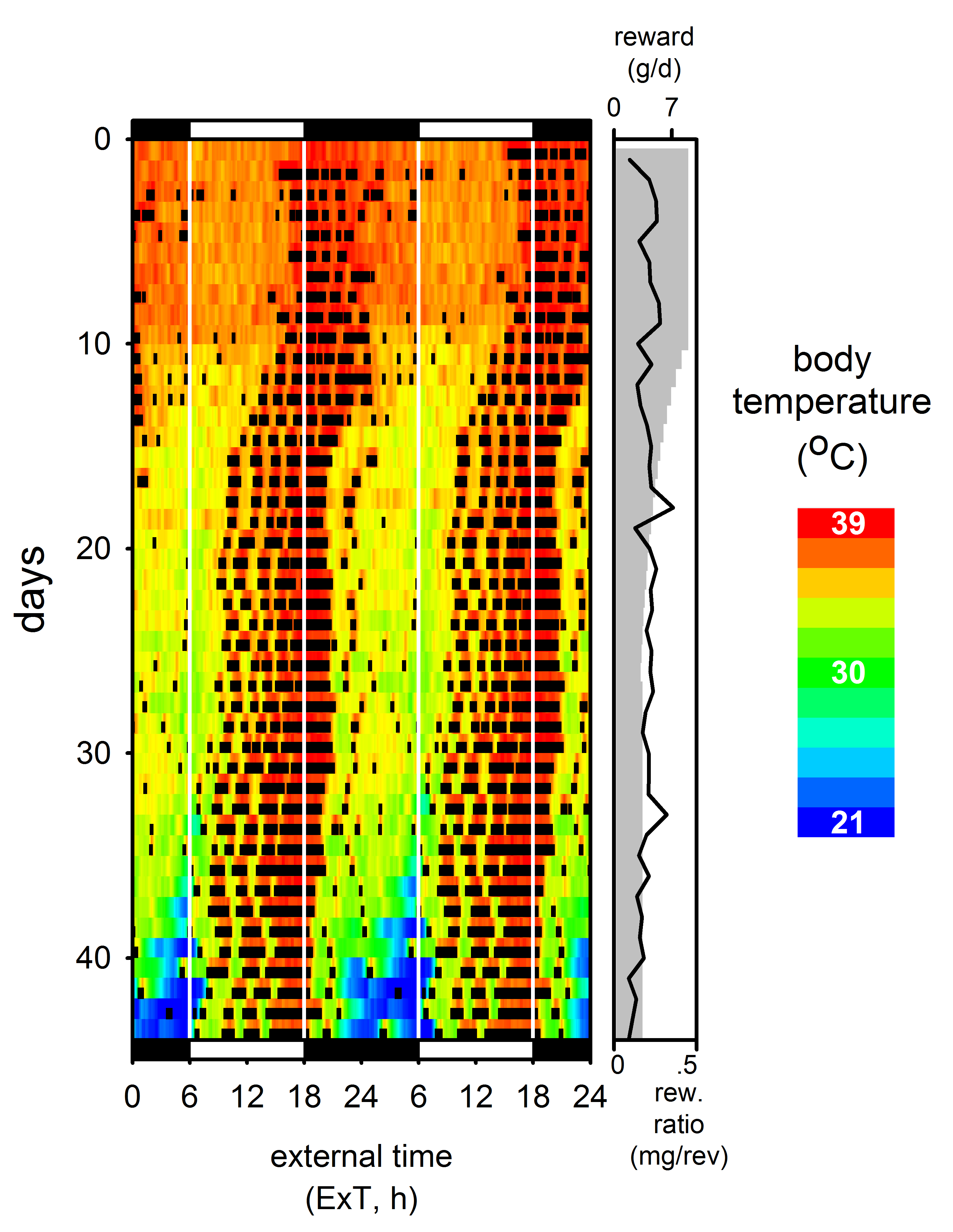

Supplement: Figure S2 — Reduced food reward shifts activity rhythms before torpor occurs. This example shows that some animals in the experiment presented in Fig. 2 and Fig. S1 clearly showed a shifted activity rhythm well before torpor occurs. The opposite (torpor occurs before the activity rhythm is shifted) was never observed. These data indicate that torpor is not necessary to shift the activity rhythms into the day. It is rather the shifted activity rhythms that allow torpor to occur in the dark phase. (TIF) [file pone.0017527.s003.tif]
